# Specifying how intervention content is communicated: Development of a Style of Delivery Ontology

**DOI:** 10.12688/wellcomeopenres.19899.1

**Published:** 2023-10-12

**Authors:** Alison J. Wright, Lisa Zhang, Ella Howes, Clement Veall, Elizabeth Corker, Marie Johnston, Janna Hastings, Robert West, Susan Michie

**Affiliations:** 1 Centre for Behaviour Change, University College London, London, England, UK; 2 Institute of Pharmaceutical Science, King's College London, London, England, UK; 3 Grounded Research, Rotherham Doncaster and South Humber NHS Foundation Trust, Doncaster, England, UK; 4 Clinical and Applied Psychology Unit, The University of Sheffield, Sheffield, England, UK; 5 Aberdeen Health Psychology Group, University of Aberdeen, Aberdeen, Scotland, UK; 6 Institute for Implementation Science in Health Care, Faculty of Medicine, University of Zurich, Zürich, Switzerland; 7 School of Medicine, University of St Gallen, St. Gallen, Switzerland; 8 Institute of Epidemiology and Health Care, University College London, London, England, UK

**Keywords:** ontology, intervention delivery, style of delivery, behaviour change, communication, communication style, intervention reporting, evidence synthesis

## Abstract

**Background:** Investigating and enhancing the effectiveness of behaviour change interventions requires detailed and consistent specification of all aspects of interventions. We need to understand not only their content, that is the specific techniques, but also the source, mode, schedule, and style in which this content is delivered. Delivery style refers to the manner by which content is communicated to intervention participants. This paper reports the development of an ontology for specifying the style of delivery of interventions that depend on communication. This forms part of the Behaviour Change Intervention Ontology, which aims to cover all aspects of behaviour change intervention scenarios.

**Methods:** The Style of Delivery Ontology was developed following methods for ontology development used in the Human Behaviour-Change Project, with seven key steps: 1) defining the scope of the ontology, 2) identifying key entities and developing their preliminary definitions by reviewing 100 behaviour change intervention evaluation reports and existing classification systems, 3) refining the ontology by piloting the ontology through annotations of 100 reports, 4) stakeholder review by eight behavioural science and public health experts, 5) inter-rater reliability testing through annotating 100 reports using the ontology, 6) specifying ontological relationships between entities, and 7) disseminating and maintaining the ontology.

**Results:** The resulting ontology is a five-level hierarchical structure comprising 145 unique entities relevant to style of delivery. Key areas include communication processes, communication styles, and attributes of objects used in communication processes. Inter-rater reliability for annotating intervention evaluation reports was α=0.77 (good) for those familiar with the ontology and α=0.62 (acceptable) for those unfamiliar with it.

**Conclusions:** The Style of Delivery Ontology can be used for both annotating and describing behaviour change interventions in a consistent and coherent manner, thereby improving evidence comparison, synthesis, replication, and implementation of effective interventions.

## Introduction

Behaviour change interventions vary considerably in the manner in which they are delivered. Specifying how an intervention is delivered is essential to understanding its effectiveness, its mechanisms of action, and reasons for variation in effectiveness across contexts ( Dombrowski *et al*., 2016). To facilitate better replication and implementation of effective behaviour change interventions, we need to report them clearly and with a sufficiently granular level of specification ( Michie *et al*., 2020; Michie *et al*., 2023).

The delivery of an intervention can include the following components ( Michie *et al*., 2021): 1) mode of delivery, *i.e.* the medium through which an intervention is provided, for example printed materials ( Dombrowski *et al*., 2016; Marques *et al*., 2021), 2) intervention source, *i.e.* who delivers the intervention, for example a health professional ( Norris *et al*., 2021), 3) intervention schedule, *i.e.* the temporal organisation of how the intervention is provided, for example, the duration of parts of the intervention, and 4) style of delivery, *i.e.* the manner in which the intervention content, such as behaviour change techniques ( Corker *et al*., 2023; Marques *et al*., 2023; Michie *et al*., 2013), is delivered, for example, cold and distant versus warm and accepting communication style ( Dombrowski *et al*., 2016; Hagger & Hardcastle, 2014). We consider style of delivery in this paper.

The impact of intervention content on behaviour is likely to vary depending on how it is delivered to participants, either enhancing or undermining its effectiveness. For example, the behaviour change technique ‘inform about health consequences’ could be presented in an authoritarian formal manner, or in an informal conversational manner. Additionally, certain groups of people may prefer particular delivery styles due to differences in social or cultural norms or other factors. This likely contributes to differences in effectiveness of interventions. For interventions that depend on communication, style of delivery includes the type of language used and the nature of the interactions between two or more participants ( Hagger & Hardcastle, 2014). A communication process can involve a variety of participants, such as humans interacting synchronously or asynchronously, a human talking to an artificial intelligence-based conversational agent ( *i.e.*, chatbots), or two computer systems exchanging information.

Style of delivery plays an important role in interventions targeting behaviours from a variety of domains including health, sustainability, and education. For example, motivational interviewing (MI) interventions use multiple techniques to increase motivation for behaviour change which are delivered using a client-centred interpersonal style ( Miller & Rollnick, 2012). MI aims to elicit the client’s own reasons for change, which requires the intervention source to adopt non-pressuring language and use a reflective, respectful, and empathic style ( Hardcastle *et al*., 2017). In the medical field, extensive work has been conducted on training healthcare professionals to strengthen their communication skills to enhance patient outcomes. For example, the Roter Interaction Analysis System ( Roter & Larson, 2002) provides a method to describe communication between healthcare professionals and patients. Studies using this method have found large variations in the communication profiles of healthcare professionals and that these variations may affect patient outcomes such as adherence to treatment ( Pires & Cavaco, 2014).

Reporting guidelines aim to improve the quality of intervention reporting in terms of specificity, consistency, and comprehensiveness. However, reporting of delivery style components has tended to be neglected. Widely used existing guidelines such as the Template for Intervention Description and Replication (TIDieR) guidance ( Hoffmann *et al*., 2014), and the CONsolidated Standards of Reporting Trials statement (CONSORT) ( Schulz *et al*., 2010) and its extension for social and psychology interventions (CONSORT-SPI) ( Montgomery *et al*., 2018) do not specify reporting the style in which intervention content is communicated. An attempt has been made to classify the delivery style of MI interventions ( *e.g.* engaging techniques such as open-ended questions; focusing techniques such as permission to provide information and advice) ( Hardcastle *et al*., 2017) however, there is no broader categorisation system. More work is needed to extensively specify style of delivery, in other words, the manner in which intervention content is communicated.

An ***ontology*** is one such method for specifying and classifying aspects of interventions (see Table 1 for glossary of bold, italicised ontology-related terms). Ontologies are standardised representational frameworks ( *i.e.* classification systems) providing a set of terms for the consistent description of data and information across disciplinary boundaries ( Arp *et al*., 2015). Ontologies represent information in the form of clearly expressed and unambiguous ***entities***, labels and definitions corresponding to these entities, and the ***relationships*** between the entities ( Arp *et al*., 2015; Hastings, 2017; Larsen *et al*., 2017). This provides formal specification and a ‘controlled vocabulary’ for a given discipline that can be understood across disciplines and topic domains ( National Academies of Sciences, Engineering, and Medicine, 2022). Entities are also given unique identifiers, allowing them to be computer-readable. This can support the application of artificial intelligence and machine learning-based approaches in data extraction, evidence synthesis, and outcome prediction ( Hastings, 2017). A further advantage of ontologies is that they are dynamic; they can and should be maintained and updated in response to new evidence emerging in the relevant discipline.

**Table 1.** Glossary.

Term	Definition	Source
**Annotation**	Process of coding, or tagging, selected parts of documents or other resources to identify the presence of ontology entities.	Michie *et al*. (2017)
**Annotation guidance manual**	Written guidance on how to identify and tag pieces of text from intervention evaluation reports with specific codes relating to entities in the ontology, using for example EPPI-Reviewer software.	Michie *et al*. (2017)
**Basic Formal Ontology (BFO)**	An upper-level ontology specifying foundational distinctions between different types of entity, such as between continuants and occurrents, developed to support integration, especially of data obtained through scientific research.	Arp *et al*. (2015)
**Class**	A category of entities as represented in an ontology.	Arp *et al*. (2015)
**Entity**	Anything that exists or can be imagined, including objects, processes, and their attributes. It Includes mental process, *i.e.*, the process and content of cognitive representations, and emotions. Entities can be represented hierarchically by parent and child classes (see definition of **parent class**).	Arp *et al*. (2015)
**EPPI-Reviewer**	A web-based software program for managing and analysing data in all types of systematic review (meta-analysis, framework synthesis, thematic synthesis etc. It manages references, stores PDF files and facilitates qualitative and quantitative analyses. It also has a facilitate to annotate published papers.	Thomas *et al.* (2010) EPPI-Reviewer 4: http://eppi.ioe.ac.uk/eppireviewer4/ EPPI-Reviewer Web Version: https://eppi.ioe.ac.uk/eppireviewer-web/
**GitHub**	A web-based platform used as a repository for sharing code, allowing version control.	https://github.com/
**Inter-rater reliability**	Statistical assessment of similarity and dissimilarity of coding between two or more coders. If inter-rater reliability is high this suggests that ontology entity definitions and labels are being interpreted similarly by the coders.	Gwet (2014)
**Interoperability**	Two systems are interoperable to the extent that the data in each system can be used by the other system. Note: An ontology is interoperable with another ontology if it can be used together with the other ontology.	http://www.obofoundry.org/principles/fp-010-collaboration.html
**Issue tracker**	An online log for problems identified by users accessing and using an ontology.	BCIO Issue Tracker: https://github.com/HumanBehaviourChangeProject/ontologies/issues
**Open Biological and Biomedical Ontology (OBO) Foundry**	A collective of ontology developers that are committed to collaboration and adherence to shared principles. The mission of the OBO Foundry is to develop a family of interoperable ontologies that are both logically well-formed and scientifically accurate.	Smith *et al*. (2007) www.obofoundry.org/
**Ontology**	A standardised representational framework providing a set of entities for the consistent description (or “annotation” or “tagging”) of data and information across disciplinary and research community boundaries.	Arp *et al*. (2015)
**Parent class**	A class within an ontology that is hierarchically related to one or more child classes (subclasses) such that all members of the child class are also members of the parent class, and all properties of the parent class are also properties of the child class.	Arp *et al*. (2015)
**Process**	Something that takes place over time.	Arp *et al*. (2015)
**Relationship**	The manner in which two entities are connected or linked.	Arp *et al*. (2015)
**ROBOT**	An automated command line tool for ontology workflows.	Jackson *et al*. (2019) http://robot.obolibrary.org
**Uniform Resource Identifiers (URI)**	A string of characters that unambiguously identifies an ontology or an individual entity within an ontology. Having URI identifiers is one of the OBO Foundry principles.	http://www.obofoundry.org/principles/fp-003-uris.html
**Versioning**	Ontologies that have been released are expected to change over time as they are developed and refined, leading to a series of different files. Consumers of ontologies must be able to specify exactly which ontology files they used to encode their data or build their applications and be able to retrieve unaltered copies of those files in perpetuity. Versioning is one of the OBO Foundry principles.	http://www.obofoundry.org/principles/fp-004-versioning.html
**Web Ontology Language (OWL)**	A formal language for describing ontologies. It provides methods to model entities of “things”, how they relate to each other and the properties they have. OWL is designed to be interpreted by computer programs and is extensively used in the Semantic Web where rich knowledge about web documents and the relationships between them are represented using OWL syntax.	https://www.w3.org/TR/owl2-quick-reference/

**Figure 1.**
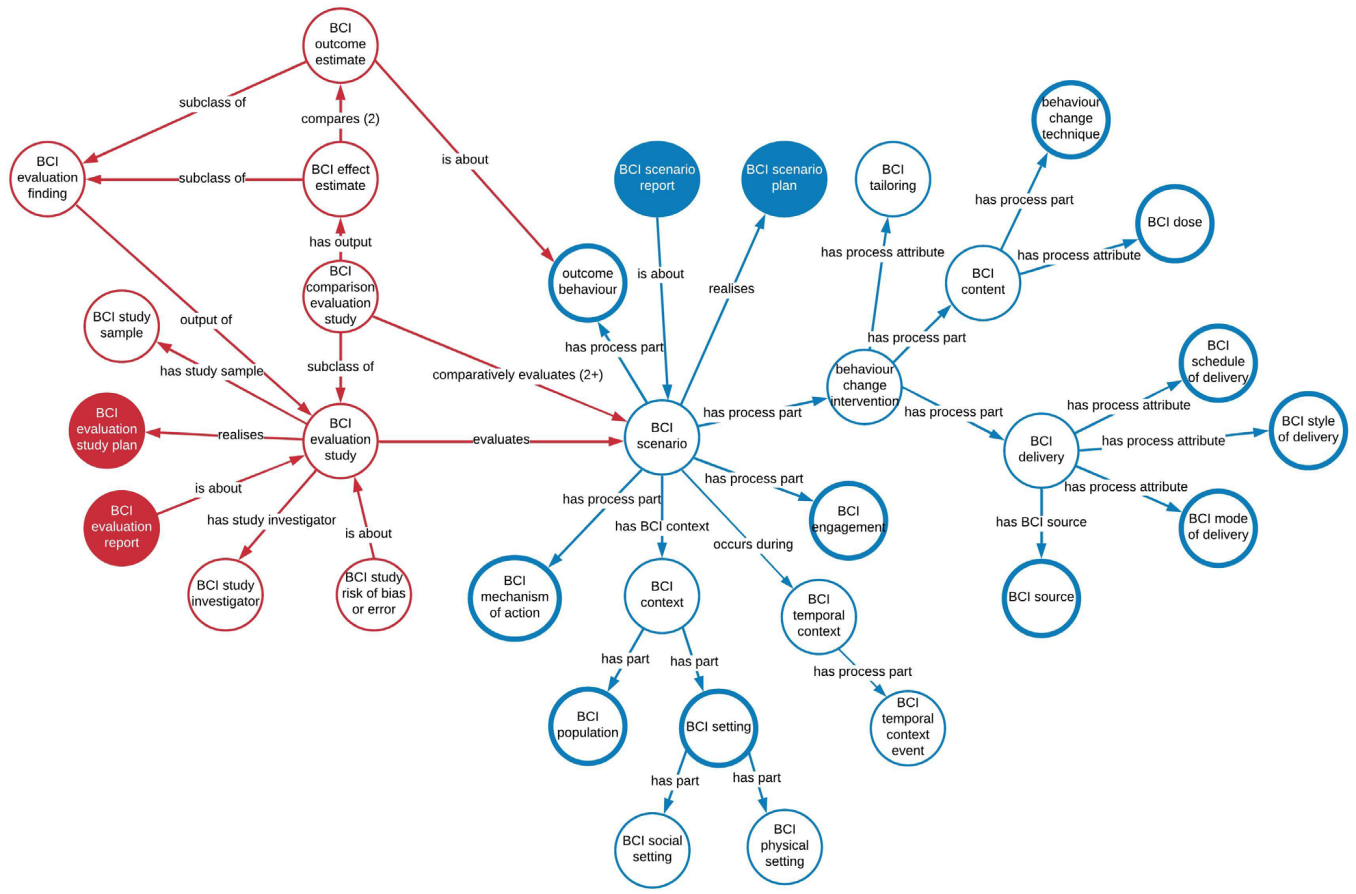
The upper level of the Behaviour Change Intervention Ontology (BCIO) V1.5.

No ontology currently exists to specify and classify the full detail of style of delivery. A comprehensive Behaviour Change Intervention Ontology (BCIO) is being developed as part of the Human Behaviour-Change Project ( Michie *et al*., 2017; Michie *et al*., 2020). The BCIO characterises behaviour change interventions, their contexts, and their evaluations. A benefit of the BCIO, compared to other classification systems such as taxonomies, is that aspects of a behaviour change intervention scenario can be linked, such as their content, delivery, mechanisms of action, target behaviours, engagement, and contextual features such as target population and setting ( Michie *et al*., 2021). The BCIO consists of an upper level with 42 entities, one of which is behaviour change intervention style of delivery, specified as ‘an attribute of a behaviour change intervention delivery that encompasses the characteristics of how a behaviour change intervention is communicated’ ( Michie *et al*., 2021). Figure 1 shows how style of delivery fits within the upper level of the BCIO.

To develop the Style of Delivery Ontology, we used the ontology development methodology specified for the Human Behaviour-Change Project ( Wright *et al*., 2020). This method was designed to meet principles of good practice in ontology development, and is a tried and tested approach for developing the lower-level ontologies of the BCIO.

## Aim

This study aimed to develop a Style of Delivery Ontology that can be reliably used to identify and specify the style in which behaviour change intervention content is delivered.

## Methods

### Ethics

Ethical approval was granted by University College London Research Ethics Committee (CEHP/2020/579). Informed participant consent was obtained in the expert stakeholder review step via an online Qualtrics survey.

### Design

The Style of Delivery Ontology was developed in an iterative process of seven steps ( Wright *et al*, 2020). Labels and definitions of each entity were written to be consistent with principles and guidelines for writing ontological definitions ( Michie *et al*., 2019; Seppälä *et al*., 2017).

### Step 1 – Development of the scope and definition of the Style of Delivery Ontology

A preliminary definition for ‘style of delivery’ and the scope of the ontology was developed by reviewing dictionaries and key papers ( *e.g.*, Dombrowski *et al*., 2016; Hagger & Hardcastle, 2014).

### Step 2 – Identifying key entities and developing the preliminary Style of Delivery Ontology

A preliminary prototype version of the ontology was developed using both a bottom-up and top-down approach. In the bottom-up approach, 100 published reports of behaviour change interventions were reviewed to develop a preliminary list of terms relevant to style of delivery. This number of reports was chosen to generate a wide preliminary range of entities for inclusion in the ontology ( Wright *et al*., 2020). These reports were part of a large dataset of behaviour change intervention reports partially annotated for behaviour change techniques, mechanisms of action, and modes of delivery, covering a range of health behaviours ( Carey *et al*., 2019; Michie *et al*., 2015). In the top-down approach, existing ontologies were searched using the list of terms created in the bottom-up approach, via specialist ontology search engines such as the Ontology Lookup Service. Terms from other ontologies were reused if the ontology used ***Basic Formal Ontology (BFO)*** as an upper level ( Arp *et al*., 2015; Smith & Grenon, 2004). If a relevant ***class*** (note that the terms ‘entity’ and ‘class’ can be used interchangeably to refer to entities represented in an ontology. For simplicity, we use the term ‘entity’ throughout) was found that was not from a BFO-based ontology, a new entity was created based on that entity but made to fit with the BFO upper level. The previous entity was cross-referenced in the new entity.

### Step 3 – Refining the ontology through literature annotation, discussion and revision

The preliminary ontology was revised by the research team based on ***annotations*** of published, peer-reviewed intervention evaluation reports. Using ***EPPI-Reviewer 4*** software ( Thomas *et al*., 2010), pairs of researchers independently annotated 100 intervention reports using the preliminary Style of Delivery Ontology. This number of papers was used as previous work has found that no additional entities are likely to become apparent for inclusion in the ontology at this point ( Wright *et al*., 2020). An open alternative to this software used for annotation is PDFAnno ( Shindo *et al*., 2018).

Reports to annotate were identified from several systematic reviews on behavioural health interventions ( Dambha-Miller *et al*., 2017; Kelley *et al.*, 2014; Makin *et al.*, 2021). These reviews were identified by using a search criterion informed by the terms identified in the bottom-up approach of entity identification. Titles and abstracts were screened from these reviews. Papers were included for annotation if: i) the outcome was a behaviour and the intervention description mentioned some aspect related to style of delivery or ii) the outcome was whether someone could be trained to use a particular style of delivery (list of papers used in the development of the ontology available on Open Science Framework).

The corpus that was used to guide the development of other ontologies in the Behaviour Change Intervention Ontology ( *e.g.*, Norris *et al*., 2021) was not used as early exploratory work found that relatively few of these reports contained information related to delivery style.

An ***annotation guidance manual*** was created to provide information on how and when to annotate each style of delivery entity. This promoted standardisation of the procedure. Discrepancies in annotations between annotators were discussed and the ontology structure, definitions and annotation guidance were revised accordingly. 

### Step 4 – Expert stakeholder review

A total of 25 expert stakeholders were invited to give feedback on the Style of Delivery Ontology resulting from Step 3. The experts came from an existing list of behavioural science and public health experts who had provided feedback on previous projects at the UCL Centre for Behaviour Change, and stakeholders who expressed interest in being involved in the Human Behaviour-Change Project stakeholder initiatives in response to invitations on Twitter and the Project newsletter. Experts were purposively selected to ensure geographic diversity of participants.

Invitations to participate were sent via email, with feedback collected through an online questionnaire using Qualtrics software. The task was designed to take no longer than 45 minutes to complete. Alongside the link to the questionnaire, experts were emailed a copy of the full ontology as a spreadsheet and in a diagram in case they found these useful when completing the task. Experts were not reimbursed for their participation.

The task presented participants with the labels and definitions of the entities in the Style of Delivery Ontology. The task asked experts to:

1. Judge whether the labels or definitions of any entities needed to be refined. If so, the experts were asked to suggest changes to these entities
2. Suggest whether any entities were missing from the Style of Delivery Ontology. If so, the experts were asked to suggest labels and definitions from these additional entities
3. Suggest whether any entities needed to be re-organised in the ontology’s hierarchy
4. Provide any other or general comments on the ontology.

### Step 5 – Inter-rater reliability of annotations using the Style of Delivery Ontology

Assessment of ***inter-rater reliability*** of the annotations by two researchers leading the development of the ontology was conducted using 50 papers. This number of papers was selected as it would give a 10–15% margin of error around the estimated percentage agreement between annotators ( Gwet, 2014; Wright *et al*., 2020). Inter-rater reliability was also assessed for annotations of a separate batch of 50 papers by two behaviour change experts unfamiliar with the ontology but with experience in annotating behaviour change intervention reports.

To identify appropriate papers to annotate, we conducted a search using Web of Science and PubMed. We used the following search terms: “communication style” or “style of delivery” or “communication skills”, combined with “intervention”. We also screened individual studies included in relevant systematic reviews in the Cochrane Consumers and Communication Group library. Studies had to include one or more of the three style of delivery-related search terms in the title/abstract to be included for full-text screening. The inclusion criteria for full-text screening was: i) quantitative studies, and ii) an experimental/intervention design ( *i.e.*, not observational). The style of delivery could be part of an intervention or assessed as the outcome of an intervention ( *e.g.*, in studies of interventions to enhance healthcare professionals’ communication skills). We selected the first 100 papers that met these criteria.

Inter-rater reliability was assessed using Krippendorff’s Alpha ( Hayes & Krippendorff, 2007) calculated using version 1.0.0 of the Automation Inter-Rater Reliability script developed by the Human Behaviour-Change Project team ( Finnerty & Moore, 2020), incorporating the python script Krippendorrff 0.3.2. The research team made additional changes to the ontology based on the issues arising from inter-rater reliability testing.

### Step 6 – Specifying relationships between Style of Delivery Ontology entities

We established relationships between entities to formally capture the knowledge present in the ontology. Relationships were specified in line with Basic Formal Ontology principles described in Arp *et al*. (2015) and the Relation Ontology ( Smith *et al*., 2005). The suitability of common relationships from these ontologies were assessed, including the basic hierarchical relationship ‘ *is_a*’ which holds between entities where one entity is a ***parent class*** of another entity.

### Step 7 – Disseminating and maintaining the Style of Delivery Ontology

The Style of Delivery Ontology was initially developed as a table of entities, with separate rows for each entity annotated with a primary label, definition, comment, synonyms, examples, and relationships. When the Style of Delivery Ontology was at a stable level of development for initial release, it was converted into ***Web Ontology Language (OWL)*** ( Antoniou & Harmelen, 2004) format, which is a standard representation format for ontologies widely used across domains. This allows it to be viewed and visualised using ontology software such as Protégé and to be compatible with other ontologies. The conversion to OWL used the ***ROBOT*** ontology toolkit library ( Jackson *et al*., 2019), which provides a facility to create well-formatted ontologies from templates. A ROBOT template is a comma-separated values (CSV) file that can be prepared easily in common spreadsheet software and annotated with instructions for translation from spreadsheet columns to OWL language and metadata attributes. Within the input template spreadsheet, separate columns represent the entity ID ( *e.g.* BCIO:044000), name, definition, comment, relationship with other entities, examples, and synonyms.

This OWL version of the Style of Delivery Ontology was then stored on the project ***GitHub*** repository, an online platform for sharing and ***versioning*** resources. GitHub has an ***issue tracker*** which allows feedback to be submitted by members of the community and addressed in subsequent releases. The Style of Delivery Ontology was also made available as part of the growing Behaviour Change Intervention Ontology within the Ontology Lookup Service ( Côté *et al*., 2006; Jupp *et al*., 2015) and the Behavioural and Social Science Ontology (BSSO) Foundry, a repository and community of practice for ontologies in the behavioural and social science domains. In addition, when all components of the Behaviour Change Intervention Ontology have been developed, it will be submitted to the ***Open Biological and Biomedical (OBO) Foundry*** ( Smith *et al*., 2007).

## Results

### Step 1 – Development of the scope and definition of the Style of Delivery Ontology

Our starting definition of style of delivery was “An attribute of intervention delivery that encompasses the characteristics of how intervention content is communicated.”

### Step 2 – Identifying key entities and developing the preliminary Style of Delivery Ontology

The preliminary version of the Style of Delivery Ontology comprised 101 entities arranged into two groups: ‘communication process’ and ‘communication style’. Communication ***processes*** are specific, directly observable communication activities ( *e.g.* asking open-ended questions) while communication styles reflect the manner in which intervention content is communicated that is not directly observable without inference ( *e.g.* empathic communication style). Communication processes and styles are related, in that the use of a particular set of communication processes contributes to a given communication style. They both relate to style of delivery when used as part of an intervention content communication process.

### Step 3 – Refining the ontology through literature annotation, discussion and revision

Annotating intervention reports led to numerous changes made to the Style of Delivery Ontology. A total of 30 entities were added, the definitions of four entities were revised, and the parent class of one entity was changed. Review by the wider ontology development group and an expert in MI led to a further nine entities being added, three removed, and changes made to the labels or definitions of 12. For example, the entity ‘positivity’ (defined as “A communication style characterised by finding optimistic aspects of the topic”) was removed because it was not sufficiently differentiable from the entity ‘communication style conveying hopefulness’ (defined as “A communication style which imparts a feeling of optimism that a person will attain a desired outcome”). At the end of this phase, the ontology contained 137 entities.

### Step 4 – Expert stakeholder review

Of the 25 experts contacted, 10 (40%) replied saying they were willing to participate in the stakeholder review of the ontology and eight (32%) completed the task. These participants were from institutions based in the following countries: Australia ( *n* = 3), England ( *n* = 2), Canada ( *n* = 1), Greece ( *n* = 1), and Brazil ( *n* = 1). All of the experts’ suggestions and how they were addressed within the ontology development process are reported in online *Extended data*.

As a result of expert feedback, two entities were added and three removed. For example, the entity ‘communication using non-word vocalisations’ was removed because experts had difficulty differentiating it from the entity ‘communication using interjections to signal attentiveness’. The labels of eight entities were revised. Updates were made to the definitions of 25 entities, with eight including a change of parent class. Many of the changes to definitions were in response to experts’ comments or queries about whether a given entity could apply to interventions where the content was delivered through digital or other means, rather than through an interpersonal interaction. We added an entity labelled ‘communication’, defined as “A process involving the exchange of information between two or more participants” to contrast with the entity now labelled ‘human communication behaviour’ and defined as “interpersonal behaviour that involves the transmission of information between two or more human beings”. Given the intention for the ontology to be broadly applicable, definitions were rewritten where appropriate to remove mention of “person”. Instead, we refer to “participants” in a communication process. Where we needed to distinguish the actions of the participant initiating or leading a particular communication process or using a certain communication style from the participant who was the target of the communication, we referred to the leading participant as the “initiator” or “instigator” and the communication target as the “recipient”. At the end of the expert stakeholder feedback review, the revised version of the ontology had 136 entities.

### Step 5 – Inter-rater reliability of annotations using the Style of Delivery Ontology

Inter-rater reliability from the 50 papers annotated by the two researchers familiar with the ontology was ‘good’ (α = 0.77; https://osf.io/dvcy6) ( Hayes & Krippendorrff, 2007). Inter-rater reliability from the 50 papers annotated by the two researchers unfamiliar with the ontology was ‘acceptable’ (α = 0.62; https://osf.io/2b874). Final revisions were made to the ontology based on issues raised by the annotators. The following changes were made: 12 entities were added, five entities were removed, and the label of one entity was updated. For example, the two entities ‘communication style conveying compassion’ (defined as “A communication style which conveys an awareness of the feelings and behaviours of another, coupled with valuing the other's welfare”) and ‘communication style conveying sensitivity’ (defined as “A communication style which empathically responds to the thoughts and feelings of the recipient”) were removed because annotators had difficulty distinguishing them from the entity ‘empathic communication style’ (defined as “A communication style which conveys an insightful awareness of the feelings and behaviours of the recipient”). Instead, we added ‘conveying sensitivity’ and ‘conveying compassion’ as synonyms of the entity ‘empathic communication style’.

### Step 6 – Specifying relationships between Style of Delivery Ontology entities

 The primary relationship used in the ontology is the *‘is_a’* relation which captures hierarchical classification. This relationship is specified for every entity in the ontology, that is, every entity is assigned a single classification parent within the ontology. In addition, the parent class ‘communication process attribute’ is related to the overarching communication process using an ‘is process attribute of’ relationship. This relationship is inherited down the hierarchy, thus, ‘intervention style of delivery’ and all its subclasses can also be inferred to be process attributes of communication processes.

### Step 7 – Disseminating and maintaining the Style of Delivery Ontology

The developed ontology consisted of 145 entities relevant to style of delivery. A downloadable version of the Style of Delivery Ontology is available from GitHub and it can be browsed in the dedicated BCIOSearch tool and diagrammatically represented using the BCIOVisualise tool. The ***Uniform Resource Identifiers (URIs)***, labels and definitions for all entities are described in Table 2. Further details ( *e.g.* comments, synonyms, examples) are found in *Extended data.* The ontology is accompanied by an annotation guidance manual that provides guidance on how to annotate for these entities in behaviour change intervention.

**Table 2.** URIs, labels and definitions for all Style of Delivery Ontology entities.

Label [URI]	Parent class	Definition
**intervention delivery [BCIO:045000]**	planned process	A planned process by which intervention content is delivered.
**behaviour change intervention delivery [BCIO:008000]**	intervention delivery	An intervention delivery in which the intervention is a behaviour change intervention.
**interpersonal behaviour [BCIO:036025]**	individual human behaviour	An individual human behaviour that involves an interaction between two or more people.
**communication [BCIO:050384]**	process	A process involving the exchange of information between two or more participants.
**intervention content communication process [BCIO:044001]**	communication	A communication that transmits the content of an intervention.
**behaviour change intervention content communication process [BCIO:044002]**	intervention content communication process	An intervention content communication process that is about behaviour change intervention content.
**communication process attribute [BCIO:044003]**	process attribute	An attribute of a communication process.
**communication style [BCIO:044004]**	communication process attribute	A particular manner of communicating aimed at inducing or avoiding certain kinds of responses in others, or demonstrating certain characteristics of the initiator.
**intervention style of delivery [BCIO:044005]**	communication style	A communication style that is an attribute of an intervention content communication process.
**behaviour change intervention style of delivery [BCIO:044000]**	communication style	A communication style that is an attribute of a BCI content communication process.
**person-centred intervention delivery [BCIO:044006]**	intervention delivery	An intervention delivery characterised by efforts by an intervention source to give a person the resources they need to manage their own life and make them an active participant in deciding how to move forward.
**participant-led intervention delivery [BCIO:044007]**	intervention delivery	An intervention delivery process in which the participant takes the lead in determining the focus of the intervention.
**source-led intervention delivery [BCIO:044008]**	intervention delivery	An intervention delivery process in which the intervention person source takes the lead in determining the focus of the intervention.
**human communication behaviour [BCIO:036034]**	inter-personal behaviour	An interpersonal behaviour that involves the transmission of information.
**linguistic communication behaviour [BCIO:050237]**	human communication behaviour	A human communication behaviour in which the information that is communicated is encoded in language.
**non-linguistic communication behaviour [BCIO:050238]**	human communication behaviour	A human communication behaviour in which information is transmitted without being encoded in the meaning units of any language.
**non-linguistic communication behaviour using body language [BCIO:050323]**	non-linguistic communication behaviour	A non-linguistic communication behaviour that involves expressing thoughts or feelings through bodily movement or posture.
**non-linguistic communication behaviour using gesture [BCIO:036002]**	non-linguistic communication behaviour using body language	A non-linguistic communication behaviour using body language involving a deliberately chosen movement of the head, neck, torso, or limbs to express an idea or feeling.
**non-linguistic communication behaviour using facial expression [BCIO:050324]**	non-linguistic communication behaviour using body language	A non-linguistic communication behaviour using body language that involves movements of facial muscles to express thoughts or feelings.
**non-linguistic communication behaviour using eye contact [BCIO:050375]**	non-linguistic communication behaviour using body language	A non-linguistic communication behaviour using body language characterised by the meeting of eyes with another individual.
**non-linguistic communication behaviour using vocalisations [BCIO:050373]**	non-linguistic communication behaviour	A non-linguistic communication behaviour using one's voice to produce sounds.
**active listening [BCIO:044009]**	communication	A communication involving one participant paying close attention to what is said by another, asking questions and reflecting as needed, in an attempt to fully understand what has been said.
**affirming communication** **[BCIO:044010]**	communication	A communication involving acknowledging another's strength or resilience in a given context or situation.
**agenda-sharing communication [BCIO:044011]**	communication	A communication in which the initiator and one or more activity participants work together to determine a focus for the activity to be undertaken.
**asking questions [BCIO:044012]**	communication	A communication in which some participant requests information from another participant.
**asking clarifying questions [BCIO:050382]**	asking questions	Asking questions in order to better understand an issue.
**asking closed-ended questions [BCIO:044013]**	asking questions	Asking questions that can only be answered with a limited set of responses.
**asking focused questions** **[BCIO:044014]**	asking questions	Asking questions designed to narrow discussion to focus on a particular aspect or topic.
**asking leading questions [BCIO:044015]**	asking questions	Asking questions designed to prompt the questionee to answer in a specific, pre-determined manner.
**asking open-ended questions [BCIO:044016]**	asking questions	Asking questions that cannot be answered with a simple yes or no, but instead require the questionee to elaborate on their points.
**banter [BCIO:044017]**	communication	A communication involving the exchange of light and teasing remarks intended to be playful.
**branded communication [BCIO:044018]**	communication	A communication using a consistent style of text or graphics to identify and distinguish a product, service or intervention from other products, services or interventions.
**communication adding intensity to another's views [BCIO:044022]**	communication	A communication using statements that add intensity to the content or emotion expressed by another person.
**communication asking permission to provide information and advice [BCIO:044023]**	communication	A communication which involves asking for approval before sharing knowledge and giving guidance.
**communication avoiding argumentation [BCIO:044024]**	communication	A communication which aims to focus on the feelings of others without debating the truth or falsity of statements or ideas.
**communication checking for others' understanding [BCIO:044026]**	communication	A communication in which the instigator asks questions to determine if the recipient has comprehended information that has been presented.
**communication checking for own understanding [BCIO:044027]**	communication	A communication in which the instigator asks questions to determine if they have correctly comprehended the information presented by another.
**communication encouraging discussion [BCIO:044028]**	communication	A communication in which the initiator promotes an extended two-way discussion regarding a topic.
**communication evoking another's ideas about change [BCIO:044029]**	communication	A communication in which the initiator attempts to elicit the recipient’s own thoughts and ideas about change rather than imposing the initiator’s own opinion.
**communication highlighting a contradiction [BCIO:044034]**	communication	A communication pointing out a situation in which actions or propositions are inconsistent with each other.
**communication inviting reactions to content presented [BCIO:044035]**	communication	A communication seeking information about a person's responses to the content presented.
**communication involving acceptance that recipients views may be different to the initiator's views [BCIO:044036]**	communication	A communication involving accepting that the recipient has different beliefs to the initiator and continuing with the planned activities while allowing both views to exist.
**communication involving agreement [BCIO:044037]**	communication	A communication in which one participant concurs with the views stated by another.
**communication involving agreement followed by highlighting incongruent aspects [BCIO:044038]**	communication involving agreement	A communication involving agreement in which one participant concurs with the views stated by another and then points out an incongruent aspect of the situation under discussion.
**communication involving confirmatory rephrasing [BCIO:044039]**	communication	A communication involving the initiator rephrasing what a recipient says in a manner intended to confirm understanding of the recipient's situation rather than confront.
**communication involving elaboration of arguments [BCIO:044040]**	communication	A communication further developing a declarative statement that has been made by making it clearer, more precise, bringing out its consequences or giving a concrete example.
**communication offering choice [BCIO:044041]**	communication	A communication involving the initiator offering a range of options to the recipient.
**communication showing acknowledgement [BCIO:050388]**	communication	A communication involving the expressed recognition of what another has expressed.
**communication acknowledging difficulties [BCIO:044021]**	communication showing acknowledgement	A communication showing acknowledgement conveying empathy or compassion towards a sub-optimal situation or concerns of a recipient.
**communication understating intensity of person's views [BCIO:044042]**	communication	A communication using statements that diminish or understate the intensity of the content or emotion expressed by the other person.
**communication using a particular form of address [BCIO:044043]**	communication	A communication addressing a person using a certain name or title to refer to that person.
**communication using formal address [BCIO:044044]**	communication using a particular form of address	A communication addressing a person using a formal, legal or official name or title to refer to that person.
**communication using informal address [BCIO:044045]**	communication using a particular form of address	A communication addressing a person using a casual or colloquial name or title to refer to that person.
**communication using preferred form of address [BCIO:044046]**	communication using a particular form of address	A communication addressing a person using a name or title that the person has specified the instigator should use.
**communication using commands [BCIO:044047]**	communication	A communication using statements that order or direct a person to think or behave in a certain way.
**communication using deliberate pauses [BCIO:044048]**	communication	A communication in which the speaker purposely leaves gaps between or after their speech utterances.
**communication using interjections to signal attentiveness [BCIO:044049]**	communication	A communication in which one person uses brief interjections to signal that they are listening to what the other person is saying.
**communication using particular language [BCIO:044050]**	communication	A communication using certain vocabulary or phraseology.
**communication using neutral language [BCIO:044051]**	communication using particular language	A communication using language in a manner which does not demonstrate an opinion on a particular issue.
**communication using non-pressurising language [BCIO:044052]**	communication using particular language	A communication using language chosen to avoid coercing or persuading the recipient to think, feel or behave in a certain manner.
**communication using slang [BCIO:044053]**	communication using particular language	A communication using informal language shared by members of a particular community.
**communication using specialist language [BCIO:044054]**	communication using particular language	A communication using words or expressions used by a profession or group that are difficult for others not in that profession or group to understand.
**communication avoiding offensive language [BCIO:044055]**	communication using particular language	A communication using language chosen by the instigator to avoid using words or phrases that could be perceived as rude or insulting.
**communication using selected words from a second language [BCIO:044056]**	communication using particular language	A communication using occasional words chosen from a language spoken by both the instigator and the recipient that is not the main language.
**communication using rational arguments [BCIO:044058]**	communication	A communication that involves the use of declarative propositions based on what the communicator believes to be logical.
**discussion [BCIO:044019]**	communication	A communication involving an extended exchange of utterances between an instigator and at least one other person.
**classroom-style discussion [BCIO:044020]**	discussion	A discussion led by the initiator in a manner typical of a teacher or lecturer.
**emotionally expressive communication [BCIO:044059]**	communication	A communication in a manner which clearly conveys the emotions of the initiator.
**exploring communication [BCIO:044030]**	communication	A communication to better understand what a person thinks or feels about a topic or an experience.
**communication exploring a person's thoughts about a topic [BCIO:050386]**	exploring communication	An exploring communication focused on what a person thinks about a matter.
**communication exploring a person's feelings about a topic [BCIO:050383]**	exploring communication	An exploring communication focused on a person's affective responses to a matter.
**communication exploring a person's thoughts about an experience [BCIO:050387]**	exploring communication	An exploring communication focused on what a person thinks about an experience.
**communication exploring a person's feelings about an experience [BCIO:050385]**	exploring communication	An exploring communication focused on a person's affective responses to an experience.
**guiding communication [BCIO:044060]**	communication	A communication that involves suggesting adoption of certain beliefs, theories or lines of action by others.
**Interrupting [BCIO:044061]**	communication	A communication in which one person interjects before another has finished making their point.
**communication avoiding interrupting [BCIO:044062]**	communication	A communication in which at least one participant endeavours to not interject before another participant has finished making their point.
**narrative communication [BCIO:044064]**	communication	A communication describing the particulars of a person's life or a course of events in the style of telling a story.
**negotiation [BCIO:044065]**	communication	A communication involving goal-oriented collaboration to arrive at an agreement or compromise on a matter of importance to the individuals involved.
**offering reassurance [BCIO:044069]**	communication	A communication intended to provide hope and strength in times of anxiety, worry, confusion, grief, distress, or suffering.
**paraphrasing [BCIO:044070]**	communication	A communication in which one participant summarises what the other said.
**reflective communication [BCIO:044071]**	communication	A communication presenting a person's previously expressed thoughts and feelings back to them.
**summarising reflective communication [BCIO:044072]**	reflective communication	A reflective communication in which the instigator pulls together key points from a complex story told by the recipient in a succinct restatement.
**double-sided reflective communication [BCIO:044073]**	reflective communication	A reflective communication in which the instigator aims to highlight both the positive and negative sides of an issue as previously expressed by the recipient.
**metaphor-using reflective communication [BCIO:044074]**	reflective communication	A reflective communication in which the instigator replaces a word or phrase of the recipient's prior assertion with a figure of speech suggesting an analogy.
**emotion-emphasising reflective communication [BCIO:044075]**	reflective communication	A reflective communication highlighting the emotional aspects of an issue as previously described by the recipient.
**re-evaluation prompting reflective communication [BCIO:044076]**	reflective communication	A reflective communication in which the instigator restates the recipient's prior assertation in a less credible way to prompt reconsideration of the assertion.
**relationship building [BCIO:044124]**	communication	A communication aimed at building or managing aspects of the interpersonal relationship between an initiator and a recipient rather than focusing on delivering communication content.
**script-based communication [BCIO:044077]**	communication	A communication which follows a pre-written sequence of instructions that are interpreted or carried out by a person or by a program.
**shifting focus of communication [BCIO:044078]**	communication	A communication that involves altering the area of greatest attention during the delivery of information.
**active participation encouraging communication style [BCIO:044079]**	communication style	A communication style marked by fostering the involvement of the recipient as an engaged actor in the activity to be undertaken rather than as a passive recipient.
**aggressive communication style [BCIO:050380]**	communication style	A communication style characterised by aggression.
**aloof communication style [BCIO:044080]**	communication style	A communication style characterised by a lack of empathy or compassion for the recipient.
**angry communication style [BCIO:050381]**	communication style	A communication style characterised by anger.
**anxious communication style [BCIO:044081]**	communication style	A communication style characterised by nervousness or unease.
**articulate communication style [BCIO:044082]**	communication style	A communication style characterised by the initiator fluently using language to express ideas.
**attentive communication style [BCIO:044083]**	communication style	A communication style characterised by purposeful consideration of another's statements.
**autonomy-supportive communication style [BCIO:044084]**	communication style	A communication style which aims to promote the recipient's capacity to make their own decisions.
**cold communication style [BCIO:044086]**	communication style	A communication style suggesting a lack of human interest or friendliness towards the recipient.
**collaborative communication style [BCIO:044085]**	communication style	A communication style characterised by a sense of the initiator and recipient working together, the initiator showing sensitivity to the others' needs, in order to obtain an outcome desired by all.
**communication style conveying acceptance [BCIO:044087]**	communication style	A communication style that conveys respect and regard for the recipient as an individual.
**communication style conveying concern [BCIO:044089]**	communication style	A communication style demonstrating one perceives an unwanted circumstance for the recipient.
**communication style conveying genuineness [BCIO:0440890]**	communication style	A communication style which suggests one is acting in line with one's true values and beliefs.
**communication style conveying hopefulness [BCIO:0440891]**	communication style	A communication style which imparts a feeling of optimism that a person will attain a desired outcome.
**communication style conveying perceived superiority [BCIO:0440892]**	communication style	A communication style which imparts a feeling that the instigator believes themselves to be higher in status than the recipient.
**communication style conveying support for change [BCIO:0440894]**	communication style	A communication style which imparts one's belief that the recipient can change.
**communication style demonstrating positive regard [BCIO:044095]**	communication style	A communication style demonstrating acceptance of everything about the recipient.
**controlling communication style [BCIO:044096]**	communication style	A communication style which directly instructs the adoption of certain beliefs, theories or lines of action.
**conversational communication style [BCIO:044097]**	communication style	A communication style that is typical of interpersonal dialogue which encourages an interactive exchange of ideas and beliefs.
**didactic communication style [BCIO:044098]**	communication style	A communication style characterised by the initiator addressing the recipient in the manner of a teacher or lecturer.
**direct communication style [BCIO:050389]**	communication style	A communication style characterised by the explicit presentation of information.
**directive communication style [BCIO:044099]**	communication style	A communication style intended to play an active role in another person's decision making.
**easily comprehended communication style [BCIO:044100]**	communication style	A communication style characterised by using language in a manner intended to be easily understandable to a recipient.
**empathic communication style [BCIO:044101]**	communication style	A communication style which conveys an insightful awareness of the feelings and behaviours of the recipient.
**engaging communication style [BCIO:044102]**	communication style	A communication style characterised by attempting to attract and maintain a recipient's interest or attention.
**entertaining communication style [BCIO:044103]**	communication style	A communication style intended to be amusing or enjoyable for the recipient.
**formal communication style [BCIO:044104]**	communication style	A communication style characterised by the use of traditional standards of correctness and etiquette and without casual, contracted, or colloquial forms of language.
**humorous communication style [BCIO:050390]**	communication style	A communication style intended to amuse.
**impatient communication style [BCIO:044105]**	communication style	A communication style conveying feelings of annoyance at time taken to understand or complete a process.
**informal communication style [BCIO:044106]**	communication style	A communication style characterised by the use of casual or colloquial language rather than formal language.
**intellectual communication style [BCIO:044107]**	communication style	A communication style characterised by an emphasis on reasoning and promoting objective understanding.
**interactive communication style [BCIO:044108]**	communication style	A communication style characterised by being responsive to the recipient's activity.
**legitimising communication style [BCIO:044109]**	communication style	A communication style characterised by making a person feel that their perspective or emotions are legitimate.
**non-confrontational communication style [BCIO:044111]**	communication style	A communication style characterised by being diplomatic and avoiding conflict.
**non-defensive communication style [BCIO:044110]**	communication style	A communication style characterised by suggesting the initiator is open to or able to accept criticism.
**non-judgmental communication style [BCIO:044112]**	communication style	A communication style that demonstrates that one is not assigning a positive or negative valence to another's views or behaviours.
**non-responsive communication style [BCIO:050391]**	communication style	A communication style characterised by not adapting to the views and behaviours of the recipient.
**patient communication style [BCIO:044113]**	communication style	A communication style conveying willingness to take time to understand or complete a process.
**persuasive communication style [BCIO:044114]**	communication style	A communication style which aims to induce or urge the adoption of certain beliefs, theories or lines of action.
**polite communication style [BCIO:044115]**	communication style	A communication style characterised by courtesy.
**relaxed communication style [BCIO:044116]**	communication style	A communication style characterised by a lack of tension or anxiety.
**respectful communication style [BCIO:044117]**	communication style	A communication style suggesting an understanding of and regard for the feelings, wishes, or rights of others.
**responsive communication style [BCIO:044118]**	communication style	A communication style characterised by adapting to the views and behaviours of the recipient.
**rushed communication style [BCIO:050392]**	communication style	A communication style conveying information in a hurried way.
**semi-structured communication style [BCIO:044119]**	communication style	A communication style involving the communication content having some constituent pre-planned parts but also being responsive to inputs.
**structured communication style [BCIO:044120]**	communication style	A communication style involving the communication content having constituent pre-planned parts.
**theatrical communication style [BCIO:044121]**	communication style	A communication style similar to that used for dramatic performance or the theatre.
**verbally dominant communication style [BCIO:044122]**	communication style	A communication style characterised by one participant having more control over a conversation than the other.
**warm communication style [BCIO:044123]**	communication style	A communication style which demonstrates human interest and caring.
**oral communication pace [BCIO:044126]**	communication process attribute	An attribute of an oral communication relating to the speed with which an individual speaks.
**tone of voice [BCIO:044125]**	communication process attribute	An attribute of an oral communication relating to the rhythm and pitch of what is said.
**readability [BCIO:044130]**	attribute	An attribute of a text used in communication process, concerning the ease of understanding the wording or other qualitative aspects of the text that facilitate comprehension.
**source visual style [BCIO:044128]**	attribute	An attribute of an intervention person source concerning how they appear to others.
**object layout [BCIO:044129]**	object attribute	An attribute of an object used in communication process, concerning how the object's components are arranged.
**object visual style [BCIO:044127]**	object attribute	An attribute of an object used in a communication process, concerning how information sensed with the eyes is presented, which may include colours/fonts/images/spacing/sizing.

## Discussion

This study developed the Style of Delivery Ontology to specify how intervention content is communicated. The resulting ontology contains 145 entities across key areas such as communication processes ( *e.g.* asking questions), non-linguistic communication behaviours ( *e.g.* using body language), communication styles ( *e.g.* empathic communication style), and attributes of objects used in communication processes ( *e.g.* visual style). Inter-rater reliability was found to be good for those familiar with the ontology and acceptable for those unfamiliar with the ontology, as assessed by Krippendorff’s alpha ( *a =* 0.77 and 0.62 respectively). This suggests that the Style of Delivery Ontology and associated annotation guidance can be applied with at least acceptable consistency.

The Style of Delivery Ontology forms part of the Behaviour Change Intervention Ontology, currently comprised of 11 other component ontologies: behaviour change techniques ( Marques *et al*., 2023), mode of delivery ( Marques *et al*., 2021), source of delivery ( Norris *et al*., 2021), setting ( Norris *et al*., 2020), mechanisms of action ( Schenk *et al*., 2023), dose (in preparation), schedule of delivery (in preparation), engagement (in preparation), fidelity (in preparation), target behaviour (in preparation), and target population (in preparation). These ontologies are connected and can be used together to answer variants of the ‘big question’: “When it comes to behaviour change interventions: What works, compared with what, for what behaviours, how well, for how long, with whom, in what setting, and why?” ( Michie *et al*., 2017). This is the overarching vision of the Human Behaviour-Change Project, and the role of ontologies is to provide organising structure to guide the application of artificial intelligence and machine learning approaches to extract and synthesise evidence, and make inferences and predictions from that evidence to generate new understanding.

Ontologies should be maintained and updated in response to new evidence about entities and their relationships ( Arp *et al*., 2015). To support ontology ***interoperability*** and limit duplication of work, ontology developers should collaborate with others where possible ( He *et al*., 2018). As with other ontologies produced as part of the Human Behaviour-Change Project ( Michie *et al*., 2020), the Style of Delivery Ontology will be refined through application and feedback from users via GitHub. In the future, updated versions of the Style of Delivery Ontology will be released via GitHub so we recommend prospective users of the ontology check there to ensure they have the most recent version of the ontology.

The Style of Delivery Ontology is applicable to a wide range of intervention content communication processes. In addition to contributing to the Behaviour Change Intervention Ontology ( Michie *et al.*, 2020), the Style of Delivery Ontology provides a stand-alone classification system for describing and reporting how intervention content is communicated with potential applications including environmental, educational, sports psychology, healthcare, and psychotherapy interventions. Such a classification system is relevant to training healthcare and other professionals to deliver intervention content in particular ways to their patients and clients by allowing for a detailed specification of the ways in which content might be communicated.

The ontology’s applicability goes beyond interventions delivered through synchronous interactions between people to also encompass a wide range of print and digital interventions, even including interventions delivered by artificial intelligence-based conversational agents, or chatbots. Its development followed calls for descriptions of interventions to go beyond simply articulating intervention content and pay greater attention to how that content is communicated ( Dombrowski *et al*., 2016; Hagger & Hardcastle, 2014).

### Strengths and limitations

A strength of this study is the use of a standardised, tried and tested method for ontology development created within the Human Behaviour-Change Project ( Wright *et al*., 2020) that reflects the OBO Foundry principles of good practice in ontology development ( Norris *et al*., 2019; Smith *et al*., 2007). As a result, the ontology fares well against several of Vrandečić's (2009) ontology evaluation criteria. In particular, the ontology’s *completeness,* or how well it covers the domain of interest, was tackled by preliminary scoping of terms from 100 papers, and annotations of a further 100 papers describing a wide range of interventions. In the stakeholder review, experts were asked if they thought any terms were missing from the ontology. The ontology’s *accuracy,* in terms of how well it accorded with experts’ knowledge, was targeted by having the ontology developed by a team of researchers with considerable experience in behaviour change interventions and by subjecting the ontology to expert stakeholder review. The ontology’s *clarity,* in terms of whether it communicated the intended meaning of the defined terms was targeted through literature annotation, where differences between annotators were considered to indicate problems with communicating meaning and the need for revisions. It was further examined through inter-rater reliability testing which indicates whether the communicated meaning of terms is sufficiently clear that annotators can agree on what constitutes an example of an entity.

A limitation of this study is that most intervention reports used in the ontology development process came from high income countries, potentially limiting the ontology’s applicability to interventions in low- and middle-income countries. While the ontology benefits from the incorporation of international expert stakeholder feedback in the ontology development process, only a third of invited experts provided feedback on the ontology. Feedback from a larger group of experts might have led to a greater range of suggested changes to the ontology. Further, our definition of ‘expert’ ( *i.e.* those with a doctoral degree in behavioural science or a related discipline) may differ to other people’s definitions, thus another group of experts from different disciplines or domains may have provided different feedback.

It is also important to acknowledge the limitations of poor reporting in intervention reports used to develop the ontology. A key part of ontology development is to identify entities from published reports during the ‘bottom-up’ phase ( *i.e.* step 2). Many reports did not describe style of delivery at all, or if they did, not in detail. This might limit the comprehensiveness of the Style of Delivery Ontology. However, it is expected that this will improve as the ontology is used more in the design and reporting of behaviour change interventions. Future applications of the Style of Delivery Ontology to a wider range of interventions and contexts will provide opportunities for it to be extended and improved.

## Conclusions

The Style of Delivery Ontology provides a classification system that can be reliably used to specify the style with which intervention content is communicated. Its use by researchers and other stakeholders will contribute to improved consistency in reporting, facilitating easier replication and simplifying evidence synthesis. The ontology will increase understanding of how the impact of intervention content ( *e.g.*, behaviour change techniques) ( Marques *et al*., 2023; Michie *et al*., 2013) varies according to the style in which such content is delivered. The Style of Delivery Ontology provides a starting point upon which future research and translation efforts can build. Its development is intended to be an ongoing and collaborative process. 

## Data Availability

Open Science Framework: Human Behaviour-Change Project. https://doi.org/10.17605/OSF.IO/EFP4X ( West *et al*., 2023). The BCIO is available from: https://github.com/HumanBehaviourChangeProject/ontologies Archived version of the Style of Delivery Ontology as at time of publication: ( https://github.com/HumanBehaviourChangeProject/ontologies/tree/master/StyleOfDelivery) Zenodo: HumanBehaviourChangeProject/ontologies: https://doi.org/10.5281/zenodo.4476603 ( Hastings *et al*., 2021) Data are available under the terms of the Creative Commons Attribution 4.0 International license (CC-BY 4.0). Open Science Framework: Human Behaviour-Change Project. https://doi.org/10.17605/OSF.IO/EFP4X ( West *et al*., 2023). This project contains the following extended data: Papers used across steps of development of the Style of Delivery Ontology ( https://osf.io/gh4xu) Version 0.1 Preliminary prototype version of Style of Delivery Ontology ( https://osf.io/s9wjg) Version 0.2 Version of Style of Delivery Ontology after initial annotations ( https://osf.io/wzdxa) Version 0.3 Version of Style of Delivery Ontology after expert stakeholder feedback ( https://osf.io/faw9q) Expert feedback survey; Full survey provided to behavioural science and public health experts in review of the Style of Delivery Ontology ( https://osf.io/rv3nu) Expert feedback on Style of Delivery Ontology: Raw feedback received from behavioural science and public health experts ( https://osf.io/r64zj) Annotation guidance manual for using the Style of Delivery Ontology ( https://osf.io/cj4zb) Internal inter-rater reliability testing ( https://osf.io/dvcy6) External inter-rater reliability testing ( https://osf.io/2b874) Style of Delivery Ontology ( https://osf.io/g7c9s) Data are available under the terms of the Creative Commons Attribution 4.0 International license (CC-BY 4.0).

## Software availability

Source code used to calculate alpha for IRR available from: https://github.com/HumanBehaviourChangeProject/Automation-InterRater-Reliability

Archived code at time of publication: https://doi.org/10.5281/zenodo.3833816 ( Finnerty & Moore, 2020)

License: GNU General Public License v3.0
