# Supplementary material for: Specifying how intervention content is communicated: Development of a Style of Delivery Ontology
Source: Wellcome Open Res. 2023 Oct 12;8:456. [Version 1] doi: 10.12688/wellcomeopenres.19899.1 (PMC11347912; doi:10.12688/wellcomeopenres.19899.1)
Supplement: Peer review history

# Reviewer report: Reviewer response for version 1

**DOI:** 10.21956/wellcomeopenres.22034.r85380

**Published:** 2024-09-04

**Authors:** Bryan A Kutner

**Affiliations:** 1 Albert Einstein College of Medicine, Bronx, USA

The authors present their process for the development of an ontology intended to allow the characterization of all aspects of the style in which behavior change interventions are delivered. This is an ambitious endeavor, the manuscript is well-written and easy to follow, and the ontology will be a useful addition to behavioral science. I intend my suggestions below to strengthen an already very clearly written article, in particular to add details that allow readers to replicate the 7-step process.

 General Comments:

 A. Among the entities in the ontology, I've not seen anything that might capture that cultural affinity between deliverer and audience might be an important aspect of Style of Delivery that influences effectiveness. For example, when peers deliver an intervention there may be shared language, experience and/or communication styles that convey the relevance of behavior change differently than a would-be credible source who is credentialed but not as closely aligned in the style of how they communicate as someone who more directly shares cultural experiences with an audience. Would the authors consider this to be an aspect of Style of Delivery?

 B. For dissemination, would the authors consider inclusion of the some of the coded excerpts that exemplify each entry, as the Human Behaviour Change Project has for the Behaviour Change Techniques Taxonomy v. 1? This would be helpful to understand how to operationalize the entities within the ontology and perhaps aid users in recognizing distinctions between entries. 

 Methods:

 A. Step 1 – Development of the scope and definition of the Style of Delivery Ontology. How did you select definitions and key papers, who reviewed these definitions and key papers, and how did you come to a final determination of the scope and definition of the Style of Delivery? These would be useful to share for those of us interested in replication.

 B. Step 2 – Identifying key entities and developing the preliminary Style of Delivery Ontology. For the bottom-down approach, how did you select 100 out of what look like more than 400 reports in the linked dataset repository? Perhaps useful as well to say "existing dataset" and to specify that these include reports through 2018 in case the linked site changes. For the top-up approach, I'd make the parenthetical here outside of the sentence itself ("If a relevant class (note that the terms ‘entity’ and ‘class’ can be used interchangeably to refer to entities represented in an ontology. For simplicity, we use the term ‘entity’ throughout) was..."). Also who conducted the searches?

 C. Step 3 – Refining the ontology through literature annotation, discussion and revision. How many pairs reviewed the 100 articles? Were discrepancies in annotations between annotators discussed just between annotators or within a larger research team (in other words, what if annotators could not come to consensus or their determinations were ambiguous)?

 D. Step 4 – Expert stakeholder review. Who resolved and how did they resolve all of the feedback from reviewers? Knowing about this process would aid replication.

 E. Step 5 – Inter-rater reliability of annotations using the Style of Delivery Ontology. Do you want to specify from among the authors who the two pairs of raters were here? Why only quantitative and experimental/interventional design studies? It seems that qualitative studies might also be informative about style of delivery. Using searches across two publication databases, how did you determine the first 100 to select? Was this organized by date or by likelihood of matching the search criteria? Readers may also want to know more about the kinds of "additional changes" as examples ("The research team made additional changes to the ontology based on the issues arising from inter-rater reliability testing").

 Results:

 A. Step 2 – Identifying key entities and developing the preliminary Style of Delivery Ontology. These definitions mirror the Motivational Interviewing Training Integrity (MITI) assessment tool that distinguishes between behavior counts and global aspects of the delivery of motivational interviewing. This may not have informed your own definitions; if it did, that lineage would be useful to share, given that you mention the involvement of one expert in motivational interviewing.

 B. Step 5 – Inter-rater reliability of annotations using the Style of Delivery Ontology. What was the experience of the two raters who were unfamiliar with the ontology in terms of their use of ontologies in general? This has implications for implementation of the ontology among 'lay' users. Related to the earlier comment on the MITI, my understanding is that inter-rater reliability for MITI counts is greater than for MITI global entities. Was this similar in your study? It would be useful for readers to see whether IRR varied among entities that fell within specific buckets (e.g., "communication processes", "non-linguistic communication behaviours", "communication styles" and "attributes of objects used in communication processes"). In general, seeing which codes had more or less IRR would also help advance where clarity might be furthered within the ontology.

 Table 1. Glossary:

 There are a few typos to correct.

 A. Basic Formal Ontology (BFO): entity->entities; please define "continuants" and "occurrents" for an audience unfamiliar with ontology development

 B. Entity: "It Includes" and is a comma intended here to distinguish a cognitive process as either cognitive or emotional? "the process and content of cognitive representations, and emotions" 

 C. EPPI-Reviewer: Check grammar

Is the work clearly and accurately presented and does it cite the current literature?

Yes

If applicable, is the statistical analysis and its interpretation appropriate?

Yes

Are all the source data underlying the results available to ensure full reproducibility?

Yes

Is the study design appropriate and is the work technically sound?

Yes

Are the conclusions drawn adequately supported by the results?

Yes

Are sufficient details of methods and analysis provided to allow replication by others?

Partly

Reviewer Expertise:

health equity in HIV services, motivational interviewing, sexual and gender minority populations, education and training of health workers, understanding behavior change techniques and mechanisms of action that mitigate HIV-related stigma in health services

I confirm that I have read this submission and believe that I have an appropriate level of expertise to confirm that it is of an acceptable scientific standard.

# Reviewer report: Reviewer response for version 1

**DOI:** 10.21956/wellcomeopenres.22034.r85375

**Published:** 2024-08-26

**Authors:** Chris Keyworth

**Affiliations:** 1 Manchester Centre for Health Psychology, Division of Psychology and Mental Health, University of Manchester, Manchester, UK

Thank you for the invitation to review this article reporting the development of a style of intervention delivery ontology. This is an interesting paper which I enjoyed reading, and will be of great value to readers across a large number of disciplines.

 Some minor suggestions are provided below:

- Intro/general rationale -  For the sentence “Additionally, certain groups of people may prefer particular delivery styles due to differences in social or cultural norms or other factors.” – An example or two might be helpful here (e.g. people with versus without a long term health conditions, or people from higher or lower socio economic backgrounds – those are just examples).
- Also in the proceeding sentences a citation or two could be provided with examples showing differences in intervention effectiveness according to delivery style. This is a key aim of the work and just a couple of citations would be helpful for added context, particularly for readers not familiar with this area.
- I like the point “A communication process can involve a variety of participants, such as humans interacting synchronously or asynchronously, a human talking to an artificial intelligence-based conversational agent ( *i.e.*, chatbots), or two computer systems exchanging information”. – I wonder if more explicit examples could be provided e.g. for humans interacting synchronously or asynchronously computer systems talking to each other.
- Methods – it might be helpful to somewhere define what the term “reports” relates to and/or includes (assuming peer reviewed publications, as well as others?) – I think this includes papers and reviews as per the link ( https://www.bct-taxonomy.com/interventions) but it might be good to explicitly define this in the text.
- Might it be helpful to list all of the specialist ontology search engines used? (Step 2 in methods).
- Good to see inter-rater reliability for the annotations – was it also done for the title and abstract screening stage?
- I think the advantages of converting into Web Ontology Language (OWL) could be a little more explicit – presumably one of the advantages is that it can potentially be integrated into IT and/or AI systems?
- I like the iterative process of developing and presenting the different versions of the ontology and looking at the links (also great to provide these) “A communication style which imparts a feeling of optimism that a person will attain a desired outcome”). At the end of this phase, the ontology contained 137 entities.” It looks like these correspond to the different versions (as well as the labels i.e. version 0.2) – you could label the version in the text more explicitly to show the iterative process. E.g. this led to version 2… etc.
- In step 7 could make it clear that the link is the final version. Might also be helpful to say it’s free to access and use with no licensing restrictions (if that is the case).
- For the sentence “Such a classification system is relevant to training healthcare and other professionals to deliver intervention content in particular ways to their patients and clients by allowing for a detailed specification of the ways in which content might be communicated.” Can you provide an example or two of the “content” of interventions.
- For the 100 papers that were used could you give a very brief description of the inclusion criteria to highlight the strength and amount of evidence reviewed (e.g. peer reviewed research studies and reviews spanning x years across x countries, although I do note the mention of high income countries in the proceeding section so you may not feel this is relevant).
- “Health professionals” and “healthcare professionals” are used interchangeably – perhaps just stick with the latter for consistency.

Is the work clearly and accurately presented and does it cite the current literature?

Yes

If applicable, is the statistical analysis and its interpretation appropriate?

Yes

Are all the source data underlying the results available to ensure full reproducibility?

Yes

Is the study design appropriate and is the work technically sound?

Yes

Are the conclusions drawn adequately supported by the results?

Yes

Are sufficient details of methods and analysis provided to allow replication by others?

Yes

Reviewer Expertise:

Behaviour change

I confirm that I have read this submission and believe that I have an appropriate level of expertise to confirm that it is of an acceptable scientific standard.

# Reviewer report: Reviewer response for version 1

**DOI:** 10.21956/wellcomeopenres.22034.r72620

**Published:** 2024-02-27

**Authors:** Ayeshah Syed

**Affiliations:** 1 University of Malaya, Kuala Lumpur, Federal Territory of Kuala Lumpur, Malaysia

The authors describe the process by which they have developed an ontology related to how behaviour change interventions are performed in communication, presenting the preliminary version of the Style of Delivery Ontology in the paper. The development of this ontology is part of a larger effort to develop a Behaviour Change Ontology encompassing all elements of behaviour change scenarios.  The research efforts focus on a much-needed area, where the numerous elements involved in behaviour change interventions as well as the diversity of understandings of what each element encompasses hinders meaningful discussion and comparison of the effectiveness of health behaviour change interventions.

 The authors provide a clear explanation of how they developed the ontology, supported by supplementary materials accessible via embedded links. In my opinion, the details provided, clear presentation, supporting tables and figures and additional documentation accessible to readers sufficiently support their description and conclusion, thereby validating the resulting ontology as being potentially useful for researchers interested in the area of behaviour change communication.

 As a qualitative researcher, however, I am not able to comment on the statistical process used to assess inter-rater reliability (Krippendorff’s Alpha, pg 7). Another point I would like to highlight is that the ontology separates communicative practices into discrete units, which is necessary for interventional study designs, while actual conversations, including those focused on  behaviour change, are interactive and collaboratively accomplished. Moreover, in the ontology itself, some of the communicative practices listed refer to specific interactional practices (e.g. asking closed-ended questions), which can be discretely observed and measured, while others refer to communicative events which are jointly accomplished by two or more interactants (e.g. interrupting, negotiation, classroom style discussion). In this sense, the labelling of the ontology as Style of Delivery may be a little limiting as it conveys a one-sided discrete behaviour and the ontology also includes communicative practices which are not necessarily related to 'style' (e.g. interruptions). Thus, the authors may want to acknowledge the limitations of the ontology as far as the need to reduce the collaborative nature of human conversation into discrete describable/measurable actions to be focused on in interventional research.

 In addition, interpretation of some items could be ambiguous, subjective or context-bound, particularly those related to communication style (interactive communication style, informal communication style, rushed communication style). In the long-term, therefore, it may be useful to work towards developing a supplementary database of example/transcripts from authentic communication to illustrate some of the items in the ontology.

Is the work clearly and accurately presented and does it cite the current literature?

Yes

If applicable, is the statistical analysis and its interpretation appropriate?

Not applicable

Are all the source data underlying the results available to ensure full reproducibility?

Yes

Is the study design appropriate and is the work technically sound?

Yes

Are the conclusions drawn adequately supported by the results?

Yes

Are sufficient details of methods and analysis provided to allow replication by others?

Yes

Reviewer Expertise:

doctor-patient interaction, discourse analysis

I confirm that I have read this submission and believe that I have an appropriate level of expertise to confirm that it is of an acceptable scientific standard.
